# The function of NAT10-driven N4-acetylcytidine modification in cancer: novel insights and potential therapeutic targets

**DOI:** 10.1186/s13578-025-01504-9

**Published:** 2025-11-28

**Authors:** Yi Wang, Sheng Wang, Maoyun Liu, Cheng Zhang, Zuotian Huang, Fengsheng Dai, Dewei Li, Hui Li

**Affiliations:** 1 Department of Hepatobiliary Pancreatic Tumor Center, Chongqing University Cancer Hospital, Chongqing, 400030 China; 2 Chongqing Key Laboratory for the Mechanism and Intervention of Cancer Metastasis, Chongqing University Cancer Hospital, Chongqing, 400030 China

**Keywords:** NAT10, RNA acetylation, Cancer, Epigenetics, Therapeutic target

## Abstract

N4-acetylcytidine (ac4C) is a novel RNA modification that plays important biological roles in a variety of diseases, including tumors, by regulating gene expression at the posttranscriptional level. As a currently known ac4C-modified “writing” protein, N-acetyltransferase (NAT10) affects the stability and translation efficiency of target mRNAs by changing the chemical and spatial structure of RNA, thereby acting as an oncogene and tumor suppressor gene in different tumors, highlighting its potential role as a tumor prognostic marker and therapeutic target. Research on the molecular mechanism of ac4C modification and its function in tumors continues to expand, but its action network and clinical translational application still face many challenges. This review systematically explains the molecular mechanism of ac4C modification and its biological significance in tumors and its connection with relevant signaling pathways and the immune microenvironment, focuses on analyzing the research progress of ac4C modification enzymes, and discusses its potential as a tumor target. The purpose of this study was to provide a theoretical basis and new ideas for basic research and the clinical translation of the ac4C modification in the field of oncology.

## Introduction

As important aspects of gene expression regulation, RNA modification has attracted widespread attention in the fields of molecular biology and oncology in recent years [1]. Recent studies have shown that RNA modifications are reversible [2–4], driven by “writer” enzymes for addition and counteracted by “eraser” enzymes for elimination [5]. These modifications not only participate in normal physiological processes but also play a key role in disease development, especially in tumor occurrence, progression and drug resistance [6, 7]. Abnormal regulation of RNA modifications has been shown to be closely related to the malignant phenotypes of multiple tumor types and has become a cutting-edge field in tumor biology research [8–10].

RNA modifications include N6-methyladenosine (m6A) and 1-methyladenosine (m1A) modifications of adenosine, 5-methyluridine (m5U) and 3-methyluridine (m3U) modifications of uridine, N7-methylguanosine (m7G) modifications of guanosine, and 5-methylcytosine (m5C) and 5-Formylcytosine (f5C) modifications of cytidine [5, 7, 11]. Among the many types of RNA modifications, N4-acetylcytidine (ac4C), a highly evolutionarily conserved modification, has been confirmed to exist widely in tRNAs, rRNAs and mRNAs in prokaryotic and eukaryotic organisms in recent years [5, 7, 11]. The ac4C modification, which has unique biological functions, is catalyzed mainly by N-acetyltransferase 10 (NAT10) through its acetyltransferase activity. It is the only known ac4C “writing enzyme”. Ac4C modification can significantly improve the stability and translation efficiency of RNA molecules, regulate the expression of target genes, and subsequently affect various life activities, such as cell proliferation, differentiation, and apoptosis [8–10]. Studies in recent years have also shown that ac4C modification is closely related to DNA repair and genomic stability. Its imbalance can lead to cell cycle abnormalities, gene expression disorders and apoptosis disorders [12].

In the majority of cases, ac4C modification is catalyzed by NAT10 or its homologous proteins. In 2014, scientists reported that NAT10 facilitates the catalysis of ac4C formation at position 1842 of 18S rRNA in human HEK293 cells [13]. Moreover, researchers reported that the Kre33 gene, a counterpart of NAT10, helps *Saccharomyces cerevisiae* produce ac4C at the 1773 site of 18S rRNA [14]. In 2015, NAT10, together with THUMPD1 and snoRNA, subsequently catalyzed the synthesis of ac4C in human HCT116 cell tRNA and 18S rRNA. The binding partners for THUMPD1 and snoRNA are tRNA and 18S rRNA, respectively [15]. Furthermore, in 2024, Zhang et al. reported that NAT10/THUMPD1 also acetylates primary microRNAs (pri-miRNAs) with ac4C modifications [16]. These findings indicate that NAT10 is an important writer of ac4C.

With an in-depth understanding of the epigenetic regulatory mechanisms of tumors, RNA modifications, especially the ac4C modification, have gradually become an emerging hotspot in tumor research and treatment [1, 7]. Studies have confirmed that NAT10 and its associated ac4C modification are highly expressed in various tumors; promote the proliferation, metastasis, drug resistance and immunosuppression of tumor cells; and are closely related to the poor prognosis of patients [17–19]. In addition, inhibitors targeting NAT10 (such as remodelin and panobinostat) have shown significant antitumor activity in vivo and in vitro, suggesting that ac4C modification is expected to become a new molecular target for tumor treatment [20, 21].

In summary, this review systematically reviews the research progress on N4-acetylcytidine modification in tumors, focusing on analyzing its molecular mechanism in tumorigenesis and development and its application prospects as a potential therapeutic target. The integration of the latest basic and clinical research results provides a theoretical basis and practical guidance for the precise diagnosis and treatment of tumors and new drug development.

## Ac4C acetylation composition, characteristics, and function

### Chemical structure and composition of ac4C

The chemical modification ac4C, with the formula C11H15N3O6, has been newly discovered in the nucleic acids of eukaryotic and prokaryotic organisms. It is deemed a key factor that modulates the efficiency and stability of mRNA translation [22]. As the initially discovered acetylated nucleoside, it is prevalent on mRNA, following m7G, m6A, and f5C in quantity [23], and plays a pivotal role in coding accuracy for protein synthesis [24].

Ac4C was first discovered in bacterial tRNA^met^ anticodons [25]. It subsequently detected on tRNA serine and leucine and 18S rRNA in eukaryotic RNA and is catalyzed by NAT10 and its homologous protein [15, 26, 27]. In 2016, researchers employed mass spectrometry to discover the presence of ac4C on human mRNA [28]. In 2018, researchers discovered a significant amount of ac4C mRNA in human cervical cancer (CESC) (HeLa) cells. In human HeLa cells, the coding sequences (CDS) region is where ac4C primarily accumulates and sequentially decreases from the 5ʹ-untranslated region to the 3ʹ-untranslated region (3' UTR) of gene transcripts [24]. However, research in 2024 slightly differed from the results reported here, with ac4C predominantly situated in the CDS, stop codon (StopC), and 3' UTR [29]. Ac4C is found in more than 4000 regions of the human transcriptome. In 2019, Tardu et al. reported high levels of ac4C in yeast mRNA samples, which significantly increased under oxidative stress conditions [23]. Additionally, Rra1 (NAT10 homolog) was found to promote the formation of ac4C mRNA. Recent findings indicate that NAT10 is responsible for catalyzing the ac4C modification of mRNAs, which is predominantly enriched in the CDS and moderately enriched in the 5' UTR [24].

### Biological characteristics of ac4C modification

**Fig. 1:**
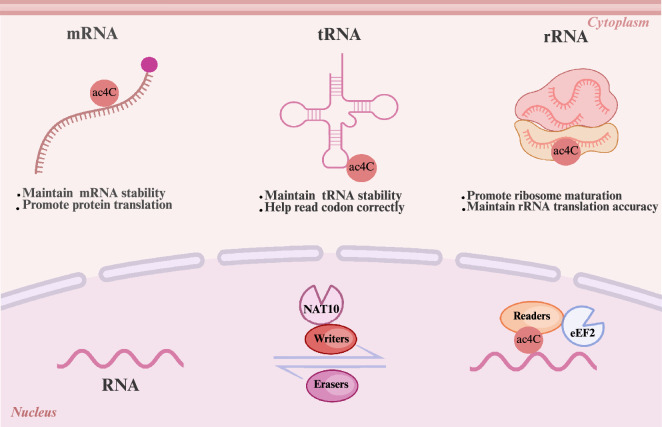
Schematic representation of N4-acetycytidine modification. The diagram illustrates the enzymatic process through which NAT10 acetylates RNA to form ac4C, while eEF2 mediates the recognition and binding to ac4C-modified sites. This modification occurs across various RNA species, such as messenger RNA (mRNA), transfer RNA (tRNA), and ribosomal RNA (rRNA). Key functional outcomes are emphasized: in mRNA, ac4C enhances stability and promotes translation efficiency; in tRNA and rRNA, it facilitates proper structural folding and functional integrity. Through these coordinated mechanisms, ac4C contributes to the regulation of essential biological pathways

RNA epigenetic modifications are typically mediated by three different types of proteins that are responsible for “writer” (deposition of specific catalytic modifications), “eraser” (elimination of particular modifications), and “reader” (recognition and binding of nucleotides with specific modifications) functions (Fig. 1). These proteins play crucial roles in influencing the fate of RNA [30]. In vivo, RNA modifications undergo dynamic changes, allowing cells to swiftly respond to environmental signals and adapt to fluctuating microenvironments caused by stress or chemotherapy treatment [31, 32]. Until 2024 September, NAT10 was the only identified protein responsible for catalyzing the formation of acetylated modified ac4C, and there is a paucity of studies on the “erasers” and “readers” involved in this modification. However, a paper published in Cancer Communication identified the “reader” protein eukaryotic elongation factor 2 (eEF2), which facilitates its binding to the ac4C sites on the CDS of target mRNAs to increase translation [20]. Studies have shown that the potential ac4C reader NOP58 [33] and eraser NAD-dependent protein deacetylase sirtuin-7 (SIRT7) are involved in RNA acetylation in snoRNA function and pre-rRNA processing [34].

### Functional roles in RNA regulation

Ac4C modifications have been found to have distinct effects on different RNAs. For example, ac4C modifications on tRNAs increase the precision of protein translation and sustain heat tolerance in organisms. In the case of 18S rRNA, ac4C is crucial for precursor rRNA processing and ribosome synthesis. It can also interact with mRNAs or tRNAs by creating a base-modified environment at the 3' end of 18S rRNA, thereby influencing translational ability [15]. Furthermore, the acetylation of mRNAs with ac4C has been determined to impact RNA structure, thereby improving mRNA stability and enhancing protein translation efficiency.

Ac4C exerts a substantial influence on gene expression regulation across multiple cancer types. Improving mRNA stability, for example, increases the expression of target genes. In bladder cancer (BLCA) cells, the downregulation of NAT10 expression reduces the ac4C modification of mRNA in specific regions, which in turn impairs the translation stability and efficiency of target genes such as BCL9-like gene (BCL9L), SRY-box transcription factor 4 gene (SOX4), and AKT serine/threonine kinase 1 (AKT1) [35]. In myeloma, ac4C acetylation is involved in modulating the stability and translation efficiency of centrosomal protein 170 (CEP170) mRNA, thus promoting its expression [36]. Furthermore, ac4C has additional diverse roles. This chemical element has been recognized as unique in the assembly of nucleic acids and plays a crucial role in regulating protein interactions and overall activity [37]. The underlying mechanisms and functions of ac4C in these processes are currently under active investigation.

### Detection and quantification of ac4C modifications

The detection and quantification of ac4C modifications are fundamental to elucidating their mechanistic roles in tumorigenesis and cancer progression. Current ac4C detection technologies primarily include high-throughput sequencing, mass spectrometry, immunoaffinity-based enrichment, chemical labeling methods, and a variety of bioinformatic prediction tools. Among these methods, high-throughput sequencing coupled with immunoprecipitation represents the mainstream approach for studying the distribution and abundance of ac4C. This method utilizes specific antibodies to enrich ac4C-containing RNA fragments, followed by deep sequencing to achieve transcriptome-wide mapping and quantitative analysis. However, antibody-dependent approaches face challenges related to specificity and sensitivity and are susceptible to background noise in complex tumor samples [38, 39].

To overcome the limitations of antibody-based strategies, several antibody-free chemical sequencing methods have been developed in recent years. For example, FAM-seq (fluorine-assisted metabolic sequencing) employs metabolic labeling with fluorine-modified analogs and subsequent chemical conversion, integrated with high-throughput sequencing, to achieve highly sensitive detection of ac4C. This technique also enables whole-transcriptome ac4C profiling in cancer cell lines [18]. Additionally, methods such as chemical reduction and the induction of C:T mismatches in sequencing (RedaC:T-seq) [40, 41] and reduction to tetrahydro-ac4C and reverse transcription with amino-dATP to induce C:T mismatches (RetraC:T) [41] leverage chemical reduction and reverse transcription-based misincorporation strategies to detect ac4C sites at single-base resolution, significantly improving quantification and localization accuracy [40, 41]. Moreover, mass spectrometry is widely used for absolute quantification of global ac4C levels, and is particularly suitable for comparative studies across tumor tissue samples [42].

In oncological applications, these techniques have been extensively employed to assess ac4C levels and their correlation with NAT10 expression in various cancers, including gastric, lung, and ovarian carcinomas, revealing functional roles for ac4C in promoting tumor cell proliferation, migration, and immune evasion [43–45]. Nevertheless, practical challenges remain, such as tumor tissue heterogeneity, RNA degradation, and low modification stoichiometry, all of which can compromise detection sensitivity and specificity. Furthermore, antibody-based methods may exhibit considerable batch-to-batch variability, chemical approaches often require stringent experimental conditions and high sample quality, and mass spectrometry demands high-purity RNA in sufficient quantities while lacking single-site resolution [22, 42].

Recent advances in artificial intelligence and deep learning have facilitated the development of bioinformatic prediction tools—such as iRNA-ac4C [46], DPNN-ac4C [47], Caps-ac4C [48], and ERNIE-ac4C [49]—that leverage sequence features to predict ac4C sites. These tools serve as valuable complements to high-throughput experimental data by improving the efficiency and accuracy of ac4C prediction [46–49]. Although highly useful in scenarios with limited samples or restricted experimental conditions, their predictions still require experimental validation.

In conclusion, while the methodological landscape for detecting and quantifying ac4C modifications continues to mature, its application in cancer research still faces multiple challenges pertaining to sensitivity, specificity, quantitative accuracy, and data interpretation. Future efforts integrating multimodal detection technologies with bioinformatic approaches will be essential for the precise dissection of ac4C modifications in both basic and clinical cancer research.

## NAT10: ac4C-modified writer protein

### Structure and functionality of NAT10

Initially, NAT10 was hypothesized to be an acetyltransferase responsible for catalyzing the generation of ac4C on rRNA, tRNA, and mRNA [22]. However, NAT10 is unique compared with other enzymes in the lysine acetyltransferase (KAT) superfamily owing to its ability to recognize tRNA and helicase segments that are often found in RNA-binding proteins [31, 50]. On the basis of sequence preservation and structural resemblance, KATs can be classified into four families, namely, GNAT, p300/CBP, MYST, and Rtt109 [51]. NAT10, also referred to as hALP, is a prominent member of the GNAT family and has histone acetyltransferase activity [52]. The NAT10 protein is principally localized in nucleoli and is implicated in maintaining nuclear shape and responding to DNA damage. Its presence in nucleoli suggests its role in various cellular processes related to RNA metabolism and gene expression regulation. The exact mechanisms through which NAT10 influences nuclear shape maintenance and the DNA damage response remain elusive [53, 54].

NAT10, present in high quantities in human cells, has been identified as a lysine acetyltransferase that targets microtubules and histones and is essential for cell division [13]. Moreover, it functions as an ATP-dependent RNA acetyltransferase that facilitates the creation of ac4C at position 1842 on the terminal helix of mammalian 18S rRNA. Involved in multiple maturation events, Kre33/NAT10 contributes to the acetylation and processing of 18S rRNA and the assembly of 40S ribosomal subunits [55]. Studies utilizing RNA interference (RNAi) to downregulate NAT10 have revealed that such downregulation delays human cell growth and results in the accumulation of high levels of 30S precursors of 18S rRNA. The involvement of NAT10 in catalyzing ac4C at position 1842 is important for the processing of rRNA and the biogenesis of ribosomes [13]. In addition, the NAT10 gene is implicated in ac4C formation on mRNA, which increases translation efficiency and stability [24, 35]. Additionally, NAT10 participates in the establishment of ac4C on tRNA, contributing to the stabilization of tRNA expression [56]. In summary, NAT10 utilizes its acetyltransferase activity to function intracellularly, not only by acetylating RNA but also by acetylating proteins such as histones and tubulins [57, 58]. These diverse activities highlight the multifunctionality of NAT10 and its importance in diverse cellular processes related to RNA regulation, protein modifications, and cell division.

NAT10 is acknowledged as the only “writer” protein for ac4C and is essential in the processes of tumor metastasis and tumorigenesis. It has also been highlighted as a target for cancer intervention [59]. NAT10, an acetyltransferase involved in regulating ac4C formation, is related to the pathogenesis of numerous conditions [60], including subtypes of ovarian cancer (OC) with poor prognosis [61] and highly aggressive colorectal cancer (CRC) [62]. Earlier studies using xenograft and mouse-based models of transgenic bladder cancer revealed that both NAT10 downregulation and its absence can lead to a reduction in tumor burden [35]. In esophageal squamous cell carcinoma (ESCC) cells, overexpression of NAT10 triggers epithelial‒mesenchymal transition (EMT), thereby enhancing tumor invasion and metastasis through fibronectin-mediated filamentous foot formation. On the other hand, deletion of NAT10 markedly suppresses ESCC cell invasion, lymph node metastasis, and lung metastasis [63]. Additionally, NAT10 has been implicated in the activation of the DNA damage response [64]. Owing to its high expression in malignant tumors, NAT10 represents a promising target for tumor treatment.

These results highlight the crucial importance of NAT10 in cancer development and progression, along with its therapeutic implications in oncology. Additional investigations are needed to delineate the molecular underpinnings through which NAT10 mediates metastatic progression and oncogenesis, as well as to develop strategies to effectively target NAT10 in cancer therapy.

### Downstream targets of NAT10

#### mRNA levels

##### Cell cycle progression and proliferation

Uncontrolled cell cycle progression and aberrant proliferation are fundamental hallmarks of cancer. In colorectal cancer, NAT10 facilitates disease progression through the NAT10/KIF23/GSK-3β/β-catenin axis. In brief, it upregulates the ac4C modification of its own mRNA by binding to the 3' UTR of KIF23 mRNA, resulting in the stabilization of KIF23 mRNA. As a consequence, KIF23 protein levels increase, resulting in the activation of the Wnt/β-catenin pathway. This activation drives the transport of β-catenin into the nucleus, where it induces the transcription of target genes such as cyclin D1 and c-Myc, ultimately promoting the progression of colorectal cancer [65]. The NAT10-mediated ac4C modification gene is also involved in the negative regulation of EGFR-activated receptor activity. ERRFI1, as a feedback suppressor of EGFR and ERBB2, is regulated by NAT10 in an ac4C-dependent manner, participates in the EGFR-mediated signaling pathway, and participates in tumor development and chemotherapy resistance in CRC [66].

Notably, NAT10-mediated RNA acetylation also plays a role in CESC [67] and oral squamous cell carcinoma (OSCC) [68] progression. It enhances HNRNPUL1 [67] and MMP1 [68] mRNA stability and promotes cancer progression. Downregulation of NAT10 inhibits the proliferative, invasive, and migratory capabilities of cervical cancer [67] and oral squamous cell carcinoma cells [68]. In vivo experiments utilizing xenograft models have corroborated the carcinogenic function of NAT10. Through RNA sequencing (RNA-seq) and acetylated RNA immunoprecipitation sequencing (acRIP-seq) analysis, HNRNPUL1 was identified as a target of NAT10 in cervical cancer. Notably, NAT10 positively regulates the expression of HNRNPUL1 by facilitating ac4C modification and ensuring the stability of HNRNPUL1 mRNA [67].

In addition, NAT10 promotes the proliferation of multiple myeloma (MM) cells by acetylating CEP170 mRNA, which increases its translation efficiency. The increased CEP170 protein then interacts with NAT10 to promote chromosomal instability, thereby accelerating MM progression [36]. NAT10 facilitates lung cancer tumorigenesis by increasing serum-glucocorticoid regulated kinase 2 (SGK2) mRNA acetylation at the 3′ UTR [69]. Through Thr367 phosphorylation, SGK2 stabilizes EZH2 by inhibiting its ubiquitination. NAT10, in turn, impedes autophagic flux both by blocking autophagosome‒lysosome fusion and by suppressing the transcription of GABARAP, which is regulated by EZH2-mediated H3K27me3 [69].

The E3 ubiquitin ligase MDM2 has been identified as a target for NAT10 in GC. Specifically, NAT10 maintains the stability of MDM2 transcripts in an ac4C-specific manner, thereby promoting the proliferation and growth of GC cells. Compared with that in control tumor samples, knockout of MDM2 transcripts led to reduced ac4C modification and MDM2 downregulation. Conversely, restoring wild-type NAT10 increased the acetylation of MDM2 mRNA and increased MDM2 expression levels. These findings indicate that NAT10-mediated ac4C modification directly regulates MDM2 [43]. Considering that MDM2 targets the tumor suppressor protein p53 for proteasome degradation and functions to suppress p53 activity, a study also explored the impact of NAT10 on the p53 pathway in cell lines with wild-type p53 (AGS and BGC823). The inverse correlation between NAT10 activity and p53 abundance can be established through dual-modality inhibition (genetic/pharmacological). The protein levels of p53 were reduced with the re-expression of wild-type NAT10, but this was not the case with mutant NAT10. Interestingly, p53 mRNA levels remained unchanged despite changes in p53 protein levels with respect to NAT10 expression. This finding implies that NAT10 primarily influences p53 protein expression rather than p53 mRNA expression [43].

In addition to its well-characterized role in stabilizing mRNAs, the function of NAT10 extends into the realm of long noncoding RNAs (lncRNAs), which are powerful regulatory molecules that orchestrate gene expression networks. Notably, in breast cancer (BC), NAT10, through its role as a lncRNA (CD2BP2-DT), promotes tumor progression [70]. By mediating the ac4C modification that stabilizes CD2BP2‐DT, NAT10 increases its expression. CD2BP2‐DT subsequently enhances CDK1 mRNA stabilization by facilitating YBX1 (RBP family) liquid‒liquid phase separation [70]. NAT10 also promotes proliferation and metastasis by increasing the stability of the suppressor of inflammatory macrophage apoptosis lncRNA SIMALR [71] in NPC cells. Mechanistically, SIMALR binds to eEF1A2 (eukaryotic translation elongation factor 1 alpha 2), and enhances its endogenous GTPase activity, thereby accelerating protein synthesis; it further promotes eEF1A2 phosphorylation, which in turn facilitates the translation of ITGB4/ITGA6 to drive the malignant phenotype of NPC cells [71]. Therefore, by mediating the ac4C modification of lncRNAs, NAT10 emerges as a central player in rewiring the noncoding RNA landscape of tumors, driving malignancy through previously unexplored mechanisms.

##### EMT and tumor metastasis

In addition to promoting primary tumor growth, NAT10 is a master regulator of epithelial-mesenchymal transition (EMT) and the subsequent multistep process of tumor metastasis. In multiple types of cancer, NAT10 participates in managing the expression of genes that are downstream targets through ac4C modification, impacting cancer progression and metastasis. In ESCC, prior investigations have utilized Kyoto Encyclopedia of Genes and Genomes (KEGG) enrichment analysis to demonstrate that the Notch signaling pathway is enriched in NAT10-overexpressing ESCC cells, suggesting that NAT10 may interact with NOTCH3 and mediate its biological processes. NOTCH3 is well known to participate in cancer progression. The overexpression and knockout of NAT10 at the mRNA and protein levels were confirmed via real-time quantitative PCR (qRT‒PCR) and Western blot (WB), which in turn increased and decreased the expression level of NOTCH3, respectively. Furthermore, NAT10 G641E mutants lacking acetyltransferase activity failed to increase ac4C enrichment or stabilize NOTCH3 mRNA, indicating that NAT10 enhances the stability of NOTCH3 mRNA through ac4C modification. Additionally, the ac4C modification of NOTCH3 mRNA was determined to be a vital regulatory switch for its function in the spread of cancer [63]. Furthermore, NAT10-mediated ac4C modification of forkhead box M1 (FOXM1) in the 3’-untranslated region promoted the malignant processes of laryngeal squamous cell carcinoma (LSCC) cells [72].

A previous study revealed that in gastric cancer (GC), NAT10 directly interacts with the 3'UTR of COL5A1 mRNA to regulate its ac4C modification, thereby promoting GC metastasis and EMT. These findings identify the NAT10/COL5A1 axis as a crucial regulatory mechanism in GC progression [73]. However, it had no effect on the protein level of COL5A1 [73]. Neutrophil extracellular traps (NETs) also drive GC cell metastasis via the ac4C modification mediated by NAT10 of SET and MYND domains containing 2 (SMYD2) in GC cells [74].

NAT10 also targets genes that influence nuclear architecture and global transcription. In a recent report, the authors identified high mobility group protein B2 (HMGB2), which plays a crucial role in the metastasis and growth of HCC, as a novel NAT10-mediated downstream effector of ac4C through RNA-seq and ribo-seq analyses [20]. In prostate cancer (PCa), NAT10 enhances the stability of high mobility group AT-hook 1 (HMGA1) and Keratin 8 (KRT8) by acetylating their mRNAs, hence accelerating cell cycle progression to improve cell proliferation and enhancing EMT progression to facilitate cell migration, respectively [18].

Similarly, in clear-cell renal cell carcinoma (ccRCC), NAT10 acts through ankyrin repeat and zinc finger peptidyl tRNA hydrolase 1 (ANKZF1) to increase the nuclear localization of yes1-associated transcriptional regulator (YAP1), thereby driving tumor progression and lymphangiogenesis [75]. Furthermore, in head and neck squamous cell carcinoma (HNSCC), NAT10-mediated ac4C modification of glycosylated lysosomal membrane protein (GLMP) mRNA activates the MAPK/ERK signaling pathway, leading to the promotion of lymph node metastasis [76].

In pancreatic cancer (PDAC), NAT10 acts as an oncogene and promotes tumorigenesis and metastasis both in vivo and in vitro. Mechanistically, through an ac4C-dependent mechanism, NAT10 increases the stability of AXL mRNA, a receptor tyrosine kinase, which leads to increased AXL expression. This, in turn, enhances the multiplication and distribution of PDAC cells, thereby contributing to their carcinogenic role [77]. NAT10 also promotes ovarian cancer cell migration and invasion by mediating the ac4C modification of CAPRIN1 mRNA. Targeting CAPRIN1, which is regulated by this modification, consequently impedes these malignant functions [78]. Thus, NAT10 acts as a master molecular switch for the metastatic cascade, coordinating the distant colonization of cancer.

##### Antiapoptotic effects and survival

Evasion of apoptosis is a hallmark of cancer, enabling transformed cells to defy normal cellular suicide signals. NAT10 critically promotes this survival advantage by directly bolstering the expression of key antiapoptotic regulators, allowing cancer cells to withstand both intrinsic and therapy-induced stress. In MM, NAT10 acetylates BCL-XL mRNA, increasing its stability and promoting protein translation. This results in the upregulation of BCL-XL, which subsequently inhibits apoptosis in MM cells and activates the PI3K‒AKT pathway, thereby facilitating cell cycle progression and MM malignancy. Thus, targeting the NAT10/BCL-XL axis may be a promising therapeutic strategy for MM [79]. Similarly, NAT10 has been postulated to increase the number of carcinoma cells in HCC [17]. In HCC, cell and animal experiments have demonstrated that the capacity of liver cancer cells to metastasize and evade apoptosis is increased by NAT10 under conditions of endoplasmic reticulum stress (ERS). This is achieved through the upregulation of ac4C modification of HSP90AA1 mRNA by NAT10, which in turn stabilizes the HSP90AA1 protein and increases its expression levels. Consequently, this mechanism augments the metastatic propensity of ERS hepatoma cells while inducing lenvatinib refractoriness, a tyrosine kinase receptor inhibitor, thereby preventing apoptosis. Research has confirmed that the NAT10 and HSP90AA1 interaction has a regulatory effect on ERS-triggered metastasis and drug resistance in liver cancer cells [17]. Thus, inhibition of apoptosis is one of the core mechanisms by which NAT10 drives tumorigenesis and development.

In addition to inhibiting classical apoptosis, recent studies have revealed that NAT10 enhances cell survival by regulating ferroptosis. NAT10 expression is upregulated in both colon cancer (COAD) tissues and multiple colon cancer cell lines. Upregulated NAT10 expression is associated with dismal survival outcomes. In both colon cancer cell lines (HT-29 and LoVo), downregulation of NAT10 impaired the proliferative, migratory, invasive, tumor-forming, and metastatic abilities of these cells; conversely, excess NAT10 expression promoted these abilities. Additional investigations revealed that NAT10 plays a crucial role in stabilizing and expressing ferroptosis suppressor protein 1 (FSP1) mRNA in HT-29 and LoVo cells. These cells undergo ac4C acetylation of FSP1 mRNA, which is an epigenetic modification tied to the inhibition of iron efflux [80]. In addition, NAT10 plays a key role in COAD development by influencing the stability and iron efflux of FSP1 mRNA, indicating that it could be a new prognostic and therapeutic target for colon cancer [80]. NAT10 also inhibits sorafenib-induced ferroptosis in NPC by increasing the stability and expression level of solute carrier family 7 member 11 (SLC7A11) [81] in NPC cells. Additionally, other findings have shown that the expression of essential iron metabolism‒related genes, including MAP1LC3A, SLC7A11, GCLC, and SLC39A8, is significantly downregulated in NAT10-deficient cancer cells. Specifically, NAT10 inhibits iron efflux by selectively stabilizing the SLC7A11 mRNA transcript, thereby inhibiting ferroptosis in BC cells [82]. In summary, by fortifying the expression of antiapoptotic and ferroptosis proteins, NAT10 critically empowers cancer cells to evade programmed cell death, thereby underpinning both tumorigenesis and therapy resistance.

##### DNA damage repair

In cancer, NAT10-mediated ac4C modification enables tumor cells to repair chemotherapy- or radiotherapy-induced DNA damage, thereby conferring treatment resistance. NAT10 confers resistance to cisplatin chemotherapy in bladder cancer cells by enhancing DNA damage repair (DDR), which is regulated by AHNAK. Mechanistically, NAT10 stabilizes AHNAK mRNA by binding to it, safeguarding it from exonuclease enzymes [83]. The overexpression of NAT10 can also increase the binding affinity of NAT10 to PARP1, promote PARP1 acetylation, prolong the half-life of PARP1, and enhance its function. This collectively leads to the recruitment of more DNA damage repair-related proteins to the site of damage, facilitating DNA damage repair and ultimately promoting the survival of BC cells [84]. Consequently, NAT10-mediated enhancement of DNA repair pathways safeguards genomic integrity in cancer cells.

##### Immune reprogramming

Immune evasion is a critical hallmark of cancer progression, enabling tumors to escape host immune surveillance. Emerging evidence establishes NAT10 as a pivotal regulator of the tumor immune microenvironment, where it fosters an immunosuppressive niche by modulating the expression of key immunoregulatory molecules in cancer cells. In nasopharyngeal carcinoma (NPC), the same family of HMGB2, high mobility group box 1 (HMGB1), the direct target of dead box helicase 5 (DDX5), is upregulated by the NAT10-mediated ac4C modification of DDX5, which inhibits the T-cell immunity of CD4 + and CD8 + T cells and promotes NPC progression [85]. Therefore, the NAT10/DDX5/HMGB1 axis suppresses T-cell function in the tumor microenvironment, thereby contributing to immunosuppression in NPC [85]. NAT10-induced ac4C modification also enhances the stability and expression of YWHAH, thereby promoting the growth and migration of CRC cells [86]. YWHAH-induced CD8 + T-cell failure can help cancer cells evade immune system [86].

In addition to suppressing immune cell function, NAT10 further drives tumor progression by recruiting and polarizing M2 macrophages. In ESCC, ac4C modification by NAT10 enhances the RNA stability of the lipid metabolism-related gene fatty acid synthase (FASN) to induce macrophage M2 polarization, which can enhance the effect of PD-1 therapy [87].

Furthermore, NAT10 modulates GAS5 expression and mediates its ac4C modification in non-small cell lung cancer (NSCLC), whereas GAS5 facilitates immune cell infiltration by activating the type I interferon signaling pathway [88]. Mechanistically, GAS5 stabilized p53 by directly binding to MYBBP1A and promoting its interaction with p53. This enhanced p53-mediated IRF1 transcription, which in turn activated type I interferon signaling and increased the production of CXCL10 and CCL5 [88]. Thus, NAT10 is a pivotal regulator of immune evasion, orchestrating an immunosuppressive tumor microenvironment through its control of immune checkpoints and cytokines.

##### Chemoresistance

Chemoresistance, a major driver of therapeutic failure in oncology, is strongly promoted by NAT10, which confers broad-spectrum defense mechanisms against chemotherapeutic agents. In melanogenesis and melanoma (SKCM), suppressing NAT10 inhibits the chemoresistance of melanoma by reducing the expression of the C2H2-ZF family members DDX41 and ZNF746 [89], respectively. Similarly, in BC, NAT10, through mediating multidrug resistance protein 1 (MDR1), and breast cancer resistance protein (BCRP), promotes tumor progression [90]. NAT10 can also promote drug resistance in MM cells by mediating exportin 1 (XPO1) mRNA N4 acetylation [91]. NAT10 can increase NANOGP8 ac4C modification levels and support NANOGP8 mRNA stability in colon cancer to maintain stemness and resist chemotherapy [92]. Ultimately, NAT10 is a pivotal orchestrator of multidrug resistance, equipping tumors with a multifaceted defensive network to circumvent the cytotoxic effects of chemotherapy.

##### Metabolic reprogramming

NAT10 drives metabolic reprogramming via ac4C modification to upregulate glycolytic and lipogenic genes, enhancing biosynthesis and fueling tumor cell proliferation. In GC, by mediating ac4C deposition at the CDS-3' UTR junction of metabolizing enzyme-hexokinase 2 (HK2) mRNA, NAT10 enhances its stability. This ultimately leads to glycolytic pathway activation and thereby drives gastric tumorigenesis [93]. In addition, NAT10 can regulate glycolysis and apoptosis in NSCLC via the acetylation of α-enolase (ENO1) by ac4C, increasing cell viability and proliferation [45]. In cervical cancer, NAT10-mediated ac4C modification of FOXP1 mRNA enhances the invasion of regulatory T (Treg) cells in the immune microenvironment (TIME) of cervical cancer by promoting glycolysis and inducing the occurrence of cervical cancer immunosuppression, thus accelerating the development of cervical cancer [94]. In ovarian cancer, NAT10 promotes tumorigenesis by mediating the ac4C modification of ACOT7 mRNA [95]. The NAT10‒ACOT7 axis orchestrates fatty acid metabolism to suppress ferroptosis, thereby promoting tumor progression [95]. In addition, in BC, NAT10, through the upregulation of JunB expression, contributes to increased glycolysis and T‑cell inhibition [44].

**Fig. 2:**
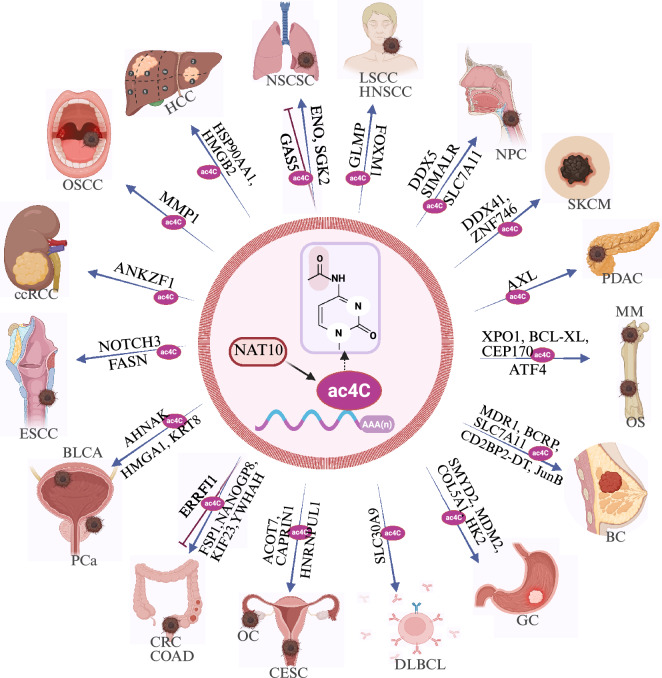
Mechanisms by which NAT10 plays a role in disease progression via RNA ac4C modification. NAT10 catalyzes the addition of ac4C marks on target RNAs, which enhances mRNA stability and translation, leading to the overexpression of proteins. Consequently, these alterations drive key disease hallmarks such as sustained proliferation, evasion of cell death, and metastasis, ultimately promoting tumorigenesis and other pathological processes

Posttranscriptional modifications also play pivotal roles in the tumorigenesis of diffuse large B-cell lymphoma (DLBCL) [96]. NAT10 regulates the mRNA stability of solute carrier family 30-member 9 (SLC30A9) in an ac4C-dependent manner; therefore, SLC30A9 promotes DLBCL cell growth by suppressing the AMP-activated protein kinase (AMPK) pathway, thereby activating the mTOR1 pathway to drive cell proliferation [96]. A recent report in osteosarcoma (OS) revealed that NAT10 enhances the stability of activating transcription factor 4 (ATF4) mRNA through ac4C modification, upregulates asparagine synthetase (ASNS) expression, and facilitates tumor cell proliferation and metastasis through Asn-mediated protein and nucleic acid synthesis [97]. In essence, NAT10 remodels the tumor metabolic landscape by increasing the flux through glycolytic and biosynthetic pathways, thereby fueling the anabolic demands of rapid proliferation (Fig. 2, Table 1).

#### Protein levels

NAT10 not only regulates the acetylation of RNA but also acetylates proteins. A study undertaken in 2016 revealed that NAT10 interacts with the p53 and Mdm2 proteins both in vitro and in vivo. Through its acetyltransferase activity, NAT10 acetylates p53 at the K120 site. Additionally, its E3 ligase activity promotes the degradation of Mdm2 proteins. These actions neutralize the inhibitory impact of Mdm2 on p53, leading to stabilized p53 levels. P53 is famously called the guardian of the genome and is important for maintaining intact genomic stability by eliminating damaged cells through either inducing cell cycle arrest for DNA repair or triggering apoptosis in response to stressful stimuli. NAT10 relocates to the nucleoplasm following DNA damage, initiating p53-mediated control of cell cycle progression and apoptosis, thereby inhibiting the proliferation of human CRC cells [98]. In hepatoma carcinoma (HCC), the level of NAT10 expression has also been shown to be positively correlated with p53 protein levels. NAT10 also increases the level of mutant p53 and promotes the proliferative ability of hepatoma cells by counteracting the role of Mdm2 in regulating p53 function [99]. In addition, NAT10 can specifically upregulate the expression of the nucleic acid acetyl coenzyme A (acetyl-CoA) to drive chemoresistance in hepatocellular carcinoma through the acetylation of ATP citrate lyase (ACLY) at K468, and K468 is required for the nuclear localization of ACLY [100]. In summary, acting as a lysine acetyltransferase, NAT10 directly acetylates a growing list of protein substrates—including p53 and ACLY—to modulate their stability, activity, or localization, thereby exerting profound effects on cell cycle progression, metabolic reprogramming, and the DNA damage response.

### Regulation of NAT10 expression

**Fig. 3:**
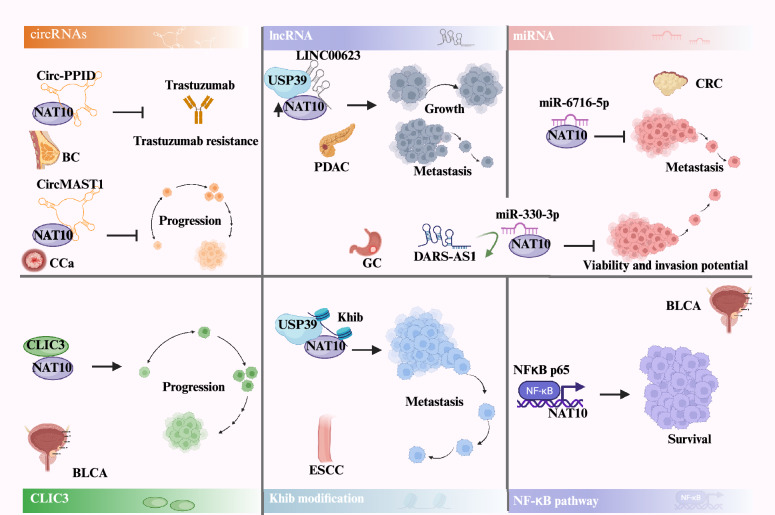
Mechanisms of upstream regulation affecting NAT10 in cancer. This schematic summarizes the multi-level regulatory network controlling NAT10 expression and activity in cancer. Upstream regulation occurs at different tiers: transcriptional level by transcription factors; post-transcriptional level through miRNAs or lncRNAs that target NAT10; post-translational level by Khib modification that affect NAT10 protein stability. This intricate network ultimately converges to dysregulate NAT10, driving oncogenesis through aberrant ac4C modification

In eukaryotes, NAT10 is responsible for catalyzing nearly all ac4C modifications in mRNA and is the only identified acetyltransferase involved in ac4C modification in mRNA [101]. NAT10 has some regulatory effects on the progression of RNA modification. A category of noncoding RNAs, lncRNAs, are those that are more than 200 nucleotides long and principally regulate gene expression through epigenetic modifications, transcriptional regulation, and posttranscriptional modifications [102, 103]. In 2022, Zengyu Feng et al. detected a novel lncRNA termed LINC00623, which enhances the tumorigenicity and migratory capacity of PDAC cells both in vitro and in vivo [101] (Fig. 3). Moreover, it promotes the growth and aggressiveness of PDAC cells by binding to NAT10 and recruiting members of the deubiquitinase protein family, specifically USP39. This interaction hinders the ubiquitination-dependent degradation of NAT10, thus increasing its protein stability. Ubiquitination plays a central role in protein degradation, suggesting a mechanism by which LINC00623 modulates NAT10 levels to promote PDAC progression [101] (Table 2).

Aberrant expression of the lncRNA DARS-AS1 has also been implicated in the progression of multiple human cancers. DARS-AS1 upregulates the expression of NAT10 by competitively binding to miR-330-3p, thereby impairing the viability and invasive potential of gastric cancer cells [104]. Conversely, miR-6716-5p binds specifically to the 3' UTR of NAT10 mRNA, leading to reduced levels of NAT10 mRNA and downregulation of NAT10 expression, eventually suppressing colorectal cancer metastasis [105], which seems to contradict the earlier identified role of NAT10 in promoting cancer growth [101].

In addition to long noncoding RNAs, circular RNAs (circRNAs) are also regulators of the ability of NAT10 to catalyze ac4C modifications. Peptidylprolyl isomerase D circular RNA (Circ-PPID), a type of circular RNA, directly interacts with NAT10 in the nucleus, preventing NAT10 from binding to human epidermal growth factor receptor 2 (HER2) mRNA, which decreases ac4C modification on HER2 exon 25 [106]. CircMAST1 competitively sequesters NAT10 and blocks Yes-associated protein (YAP) ac4C modification, thereby promoting its degradation and suppressing tumor progression in cervical cancer [107].

In a study carried out by Long Liao et al. proteomic analysis and CRISPR/Cas9 functional screening identified NAT10 as a crucial substrate for 2-hydroxyisobutylation (Khib) modification, and its role in tumor metastasis was investigated. Specifically, Khib modification at the lysine 823 site of NAT10 was shown to play a functional role in promoting the metastasis of ESCC cells. This modification enhances the interaction between NAT10 and USP39, leading to improved stability of the NAT10 protein [63].

In bladder cancer, cisplatin-induced ac4C modification has been associated with chemotherapy resistance. The activation of the NF-κB pathway by cisplatin enhances transcription by facilitating p65 binding to the NAT10 promoter. Cisplatin-induced upregulation of NAT10 expression necessitates NF-κB signaling activation, with NF-κB p65 directly binding to the NAT10 promoter to drive transcription [83]. Furthermore, both H_2_O_2_ and cisplatin can upregulate NAT10 gene expression by stimulating the transcriptional activity of the NAT10 upstream sequence from −615 to −306 bp. This region contains four tandem TATA boxes, and treatment with genotoxic drugs such as H_2_O_2_ and cisplatin leads to elevated levels of NAT10 protein and gene expression [64]. Chloride intracellular channel 3 (CLIC3) interacts with NAT10 and inhibits its function to promote bladder cancer progression, resulting in the downregulation of ac4C modification and the stability of p21 mRNA [108]. NAT10 also plays a modulatory role in mRNA stability by recruiting HNRNPQ and THRAP3 to enhance ac4C modifications adjacent to the stop codon. Knocking down HNRNPQ or THRAP3 significantly inhibits the expression of NAT10 target mRNAs [83]. In non-small cell lung cancer, c-myc-mediated upregulation of NAT10 promotes the proliferative and migratory abilities of non-small cell lung cancer cells [109]. Further investigation is essential to identify the precise mechanisms and binding sites involved in the interaction between upregulation regulators and NAT10.

### NAT10 acts as an RNA cytosine acetyltransferase for ac4C in cancer

The acetylation of RNA, specifically ac4C, has been shown to play a significant role in the regulation of gene expression in various types of cancers [60]; NAT10, the sole acetyltransferase that catalyzes the conversion of cytosine to N4-acetylcytidine, also contributes to the regulation of cancer initiation and metastasis [60, 63]. Additionally, NAT10 is involved in preserving RNA stability and enhancing translation efficiency through its RNA acetylation activity [24, 110, 111]. Earlier studies have demonstrated that NAT10 can drive tumor metastasis and drug resistance through its acetyltransferase activity [55]. In liver cancer and colorectal cancer, NAT10 has been found to induce cancer cell proliferation [98, 112]. Moreover, as an ac4C-modified acetyltransferase, NAT10 is implicated in several other cancers, including pancreatic cancer [77], stomach cancer [43], bladder cancer [35, 83], and cervical cancer [67]. In these cancers, NAT10 promotes tumorigenesis, metastasis, and chemotherapy resistance in an ac4C-dependent manner [35, 43, 67, 77, 83]. It enhances the proliferative, invasive, and migratory capabilities of cancer cells, thereby contributing to the progression and aggressiveness of the disease [65].

NAT10 has been reported to function essentially as a promoter of cancer development and progression [73, 113]. However, previous studies inferred that NAT10 can exhibit tumor-suppressive properties in certain types of cancer. For example, in non-small cell lung cancer, GAS5 impedes tumor development by curbing the proliferation of tumor cells. NAT10-mediated upregulation of GAS5 facilitates immune cell infiltration via the MYBBP1A-p53/IRF1/type I interferon signaling axis [88], and type I interferon signaling plays an important role in antitumor immunity [114]. These findings suggest the potential tumor-suppressive role of NAT10 in NSCLC. Similarly, enhancing NAT10 expression has been revealed to suppress the metastasis of colorectal cancer cells [105]. In the presence of DNA damage, NAT10 enhances the stability and transcriptional activity of p53 through acetylation of the p53 protein, thereby inhibiting the proliferation of cells with DNA damage [98]. In a 2018 study, statistical analysis of immunohistochemical staining revealed that the cytoplasmic and cell membrane localization of NAT10 in liver cancer tissues was associated with a poorer clinical prognosis than its nucleolar localization [115]. These findings underscore the multifaceted role of NAT10 in cancer, in which it can act either as an oncogenic promoter or a tumor suppressor depending on the cellular genetic background, substrate specificity and localization. During the temporal stage of tumorigenesis, the function of NAT10 may shift from protective (e.g., maintaining genomic stability in premalignant cells) to pro-oncogenic (e.g., driving proliferation and therapy resistance in established cancers) during disease progression. Further research is needed to comprehensively clarify the mechanisms underlying the dual role of NAT10 in cancer development and progression.

### Potential clinical applications of targeting NAT10

#### Diagnostic applications

Clinically, NAT10 overexpression has been associated with chemotherapy resistance, recurrence, and poor clinical prognosis in various types of cancer. In bladder cancer, NAT10 overexpression has been linked to chemotherapy resistance, as well as poor prognosis and clinical outcomes [83]. Likewise, in multiple myeloma, high NAT10 expression levels are associated with a dismal prognosis [79]. In pancreatic cancer, NAT10 overexpression has been speculated to confer resistance to gemcitabine [116]. On the basis of these findings, researchers have theorized that NAT10 could serve as a potential target and prognostic biomarker for cancer treatment, including multiple myeloma and squamous cell carcinoma of the head and neck (HNSCC) [79, 117]. Furthermore, NAT10 has shown promise as an early detection marker for cancer [118]. In liver cancer, significant variations in NAT10 expression were observed between tumor and adjacent healthy tissues, suggesting its potential utility as a supplementary biomarker for the diagnosis of malignant tumors [119]. In addition, membranous NAT10 positivity has been detected in the aggressive pretumor area of invasive nests in colorectal cancer, and its expression is linked to aggressive clinical behavior in this cancer type [62].

#### Therapeutic interventions

The therapeutic prospects of targeting the ac4C machinery are particularly compelling. As a new drug resistance driver, NAT10 is an important target for cancer therapy. Blocking NAT10-mediated gene-ac4C or decreasing the expression of NAT10 has the potential to alleviate tumor drug resistance or tumor progression. Pharmacological inhibition of NAT10 or CRISPR-mediated NAT10 knockout has shown efficacy in suppressing tumor growth and chemoresistance across preclinical models. In the majority of the studies reviewed, researchers have identified Remodelin as a potential chemical inhibitor of NAT10 [21, 96, 97, 113]. Remodelin specifically binds the active site of NAT10, disrupting NAT10-mediated gene regulation through direct suppression of its expression. Moreover, the mechanism by which Remodelin inhibits NAT10 in PCa cells involves the regulation of DNA replication to suppress the progression of prostate cancer [113]. Remodelin is also a promising antitumor drug to improve cisplatin therapy outcomes by inhibiting NAT10 expression [83].

Accumulating evidence from studies indicates that NAT10 is the primary target of Remodelin. In osteosarcoma, Remodelin inhibits osteosarcoma cell growth, and by conducting a structure-based virtual screen of a specific chemical library and FDA-approved drugs in a drug bank, researchers have shown that paliperidone and AG-401 can bind to NAT10 and inhibit osteosarcoma cell growth in vivo and in patient-derived xenograft (PDX) models; however, treatment with paliperidone and AG-401 does not alter NAT10 expression, in contrast to the effect seen with Remodelin [97]. In addition to Remodelin, fludarabine has also been found to be an inhibitor of NAT10 in acute myeloid leukemia [120] and ovarian cancer [95].

However, in HCC cells, Remodelin does not significantly inhibit NAT10-mediated ac4C modification [20]. On this basis, researchers have conducted structure-based virtual screening to dock FDA-approved drugs to the catalytic pocket of NAT10 to block NAT10-mediated modification. Panobinostat selectively binds to and occupies the ac4C catalytic activity domain of NAT10 (G641), hence blocking the ac4C modification of target RNA transcripts. Treatment with panobinostat markedly suppressed HCC cell lung metastasis and NAT10 expression in vivo, highlighting that it is an effective and safe lead compound that targets NAT10-mediated ac4C for potential HCC therapy [20]. Among the liver cancer chemotherapeutic drugs oxaliplatin and doxorubicin, ACLY can be acetylated by NAT10 at lysine 468, which confers resistance to chemotherapy in HCC cells and mouse xenografts. These findings demonstrate that ACLY K468-Ac drives chemoresistance in HCC. The K468 site was found to be conserved among multiple species. K468 is a key residue that is responsible for the nuclear localization of ACLY and is acetylated upon chemotherapy. Therefore, finding small-molecule inhibitors to block NAT10-mediated ACLY K468-Ac has the potential to alleviate HCC drug resistance [100]. In addition, targeting NAT10-mediated Eg5 K771 acetylation is a potential strategy for antitumor therapy [121].

#### Combination treatment strategies

NAT10 inhibitors often exhibit limited efficacy when used as monotherapies, primarily due to drug resistance or reversible growth inhibition. Therefore, combination therapy has emerged as a crucial strategy to overcome these limitations. Such as combined targeted therapy, combined immunotherapy, combined chemotherapy and radiotherapy. In CRC, NAT10 stabilizes ERRFI1 mRNA via ac4C modification. Inhibition of NAT10 with Remodelin reduces ERRFI1 expression, leading to EGFR reactivation, which counteracts the antitumor effect. Consequently, combining Remodelin with the EGFR inhibitor cetuximab results in synergistic antitumor activity both in vitro and in vivo. Additionally, since 5-fluorouracil (5-Fu) promotes the ubiquitin-mediated degradation of NAT10, a triple combination of 5-Fu, Remodelin, and cetuximab has been shown to be effective in KRAS wild-type colorectal cancer [66]. Moreover, paliperidone and AG-401 exhibit synergistic effects, significantly inhibiting tumor growth in an osteosarcoma PDX model [97]. Combination immunotherapy is also an important aspect. By reversing NAT10-mediated immunosuppression to convert “cold” tumors into “hot” ones, potentially enhancing the efficacy of immune checkpoint blockers. In ESCC cells, the most potent inhibition of tumor growth was achieved with the combination of NAT10 silencing and PD-1 blockade [87]. NAT10 inhibitors also enhance the efficacy of chemotherapy and radiotherapy by disrupting the DNA repair machinery of cancer cells, which increases the accumulation of lethal DNA damage [83].

In summary, inhibition of NAT10 via pharmacological means may be a promising strategy for the treatment of cancers [43]. Several potential candidates for inhibitors of NAT10 acetyltransferase activity have been identified, including fosaprepitant, leucal, fludarabine, and dantrolene [122]. Overall, these discoveries emphasize the clinical importance of NAT10 in cancer and its potential as a therapeutic target and a prognostic biomarker in multiple cancer types (Tables 1, 2).

**Table 1.** Downregulated targets of NAT10 acetyltransferase in cancer

Cancer type	Target	Result	References
GC	SMYD2	Promoting GC metastasis	[74]
GC	MDM2	Promoting GC proliferation, growth	[43]
GC	COL5A1	Promoting GC metastasis, EMT	[73]
GC	HK2	Promoting gastric tumorigenesis	[93]
HCC	HSP90AA1	Promoting HCC metastasis, resist apoptosis	[17]
HCC	HMGB2	Promoting HCC growth, metastasis	[20]
CRC	KIF23	Promoting CRC progression	[65]
CRC	YWHAH	Promoting CRC growth, metastasis	[86]
CRC	ERRFI1	Inhibiting CRC progression	[66]
ccRCC	ANKZF1	Promoting ccRCC progression, lymph angiogenesis	[75]
BC	MDR1, BCRP	Promoting BC progression	[90]
BC	CD2BP2-DT	Promoting BC progression	[70]
BC	SLC7A11	Inhibiting BC ferroptosis	[82]
BC	JunB	Promoting BC progression	[44]
NPC	DDX5	Promoting NPC progression	[85]
NPC	SIMALR	Promoting NPC proliferation, metastasis	[71]
NPC	SLC7A11	Inhibiting NPC ferroptosis	[81]
DLBCL	SLC30A9	Promoting DLBCL progression	[96]
PCa	HMGA1, KRT8	Promoting PCa metastasis	[18]
ESCC	FASN	Promoting ESCC growth	[87]
ESCC	NOTCH3	Promoting ESCC metastasis	[63]
PDAC	AXL	Promoting PDAC proliferation, metastasis	[77]
COAD	FSP1	Promoting COAD proliferation, metastasis, invasion, forming	[80]
COAD	NANOGP8	Promoting COAD chemoresistance	[92]
BLCA	AHNAK	Promoting BLCA survival	[83]
LSCC	FOXM1	Promoting LSCC malignant	[72]
HNSCC	GLMP	Promoting HNSCC metastasis	[76]
SKCM	DDX41, ZNF746	Promoting SKCM chemoresistance	[89]
MM	CEP170	Promoting MM proliferation	[36]
MM	BCL-XL	Promoting cell cycle progression, MM malignancy	[79]
MM	XPO1	Promoting MM chemoresistance	[91]
CESC	HNRNPUL1	Promoting CESC progression	[67]
OSCC	MMP1	Promoting OSCC progression	[68]
OS	ATF4	Promoting OS proliferation, metastasis	[97]
NSCLC	ENO	Inhibiting NSCLC apoptosis	[45]
NSCLC	GAS5	Inhibiting NSCLC proliferation	[88]
NSCLC	SGK2	Promoting lung tumorigenesis	[69]
OC	ACOT7	Promoting ovarian tumorigenesis	[95]
OC	CAPRIN1	Promoting OC metastasis, invasion	[78]

**Table 2.** The upstream regulators of NAT10

Cancer type	Regulator	Result	References
PDAC	LINC00623	Promoting PDAC growth and aggressiveness	[101]
GC	DARS-AS1	Impairing GC viability and invasion potential	[104]
CRC	miR-6716-5p	Suppressing CRC metastasis	[105]
BC	Circ-PPID	Suppressing the trastuzumab resistance of BC	[106]
CCa	CircMAST1	Suppressing CCa progression	[107]
ESCC	Khib	Promoting metastasis of ESCC cells	[63]
BLCA	NF-κB p65	Promoting the survival of BLCA cells	[83]
BLCA	CLIC3	Promoting BLCA progression	[108]

## Conclusions

In summary, RNA modifications, including acetylation, are important epigenetic modifications that affect gene expression at the posttranscriptional level in eukaryotic cells. These modifications have been implicated in the pathogenesis of various immune-related diseases, such as cancer, infection, inflammation, and autoimmune diseases [123]. Among the different types of RNA acetylation, ac4C is a unique modification that regulates mRNA stability and translation efficiency. NAT10 is currently the only known “writer” protein responsible for ac4C modification. It plays a critical role in cancer and catalyzes the formation of ac4C modifications on target gene mRNAs through its acetyltransferase activity. NAT10 involvement in cancer is strongly associated with cancer initiation, proliferation, and migration. However, other proteins or factors involved in regulating ac4C modification have not been identified thus far, and their roles in cancer remain unknown. Further research is needed to identify novel proteins or mechanisms involved in ac4C modification and their implications in cancer biology.

The emerging role of ac4C as a dynamic epitranscriptomic modifier in cancer underscores its importance in shaping tumor biology and therapeutic responses. Our synthesis of recent studies revealed that ac4C, through NAT10-mediated deposition, acts as a potent regulator of oncogenic mRNA stability and translation. However, whether ac4C cooperates with or competes with other modifications to fine-tune mRNA fate in tumors remains an open question. In a recent investigation, researchers revealed the interplay between m6A modifications and ac4C. In OC cells, IGF2BP1 enhanced the translation dynamics of NAT10 mRNA in a m6A-dependent manner. NAT10 promotes tumorigenesis by mediating the ac4C modification of ACOT7 mRNA, thereby increasing ACOT7 stability and translation [95]. METTL3-induced m6A modification also leads to the upregulation of NAT10 in ESCC cells [87].

In the future, an in-depth understanding of the molecular mechanism of ac4C modification and its interaction with tumor signaling pathways and the immune microenvironment will provide a solid foundation for the development of new antitumor drugs and promote the realization of precision medicine. The application of cutting-edge technologies such as multiomics integration, single-cell sequencing, and spatial transcriptome sequencing is expected to provide a comprehensive picture of tumor ac4C modification and promote relevant basic research and clinical transformation. Moreover, developing highly specific and efficient ac4C-targeted control tools is highly important for achieving personalized and precise treatment of tumors. As medical researchers and clinical workers, we should pay close attention to the latest developments in the field of ac4C modification, actively promote its application and transformation in tumor diagnosis and treatment, and bring more gospel to patients with tumors.

Overall, ac4C modification is a pivotal yet underappreciated mechanism of gene regulation in cancer. By driving core hallmarks such as proliferative signaling, resistance to cell death, and metastasis, the ac4C-NAT10 axis represents a crucial node in oncogenesis, positioning it as both a potent biomarker and a promising therapeutic target. Further investigations into the regulation of ac4C modification are anticipated to advance our comprehension of cancer biology and potentially open avenues for novel therapeutic interventions.

## Data Availability

Not applicable.

## Abbreviations

ac4C: N4-acetylcytidine
NAT10: N-acetyltransferase 10
m6A: N6-Methyladenosine
m1A: 1-Methyladenosine
m5U: 5-Methyluridine
m3U: 3-Methyluridine
m7G: N7-methylguanosine
m5C: 5-Methylcytosine
f5C: 5-Formylcytosine
CDSs: Coding sequences
3' UTR: 3ʹ-Untranslated region
StopC: Stop codon
BLCA: Bladder cancer
BCL9L: BCL9 like gene
SOX4: SRY-box transcription factor 4 gene
AKT1: AKT serine/threonine kinase 1
CEP170: Centrosomal protein 170
pri-miRNAs: Primary microRNAs
KAT: Lysine acetyltransferase
RNAi: RNA interference
ESCC: Esophageal squamous cell carcinoma
EMT: Epithelial-mesenchymal transition
GC: Gastric cancer
NETs: Neutrophil extracellular traps
SMYD2: SET and MYND domain containing 2
CRC: Colorectal cancer
HCC: Hepatoma carcinoma
ERS: Endoplasmic reticulum stress
qRT-PCR: Real-time quantitative PCR
WB: Western blot
PDAC: Pancreatic cancer
FSP1: Ferroptosis suppressor protein 1
DDR: DNA damage repair
BC: Breast cancer
FOXM1: Forkhead box M1
GLMP: Glycosylated lysosomal membrane protein
LSCC: Laryngeal squamous cell carcinoma
HNSCC: Head and neck squamous cell carcinoma
MM: Multiple myeloma
CESC: Cervical cancer
OSCC: Oral squamous cell carcinoma
RNA-seq: RNA sequencing
acRIP-seq: RNA immunoprecipitation sequencing
lncRNAs: Long noncoding RNAs
Circ-PPID: Peptidylprolyl isomerase D circular RNA
HER2: Human epidermal growth factor receptor 2
YAP: Yes-associated protein
Khib: 2-Hydroxyisobutylation
CLIC3: Chloride intracellular channel 3
eEF2: Eukaryotic elongation factor 2
OC: Ovarian cancer
HK2: Hexokinase 2
acetyl-CoA: Acetyl coenzyme A
ACLY: Acetylation of ATP citrate lyase
HMGB2: High mobility group protein B2
NPC: Nasopharyngeal carcinoma
SLC7A1: Solute carrier family 7 member 11
DLBCL: Diffuse large B-cell lymphoma
SLC30A9: Solute carrier family 30-member 9
AMPK: AMP-activated protein kinase
PCa: Prostate cancer
HMGA1: High mobility group AT-hook 1
KRT8: Keratin 8
FASN: Fatty acid synthase
COAD: Colon cancer
SKCM: Melanogenesis and melanoma
XPO1: Exportin 1
OS: Osteosarcoma
ATF4: Activating transcription factor 4
NSCLC: Non-small cell lung cancer
PDX: Patient-derived xenograft
FAM-seq: Fluorine-assisted metabolic sequencing
RedaC:T-seq: Chemical reduction and the induction of C:T mismatches in sequencing
RetraC:T: Reduction to tetrahydro-ac4C and reverse transcription with amino-dATP to induce C:T mismatches
SGK2: Serum-glucocorticoid regulated kinase 2
ANKZF1: Ankyrin repeat and zinc finger peptidyl tRNA hydrolase 1
DDX5: Dead box helicase 5
MDR1: Multidrug resistance protein 1
BCRP: Breast cancer resistance protein
ASNS: Asparagine synthetase
